# Supplementary material for: Unveiling the contribution of particle-associated non-cyanobacterial diazotrophs to N2 fixation in the upper mesopelagic North Pacific Gyre
Source: Commun Biol. 2025 Feb 22;8:287. doi: 10.1038/s42003-025-07542-w (PMC11846875; doi:10.1038/s42003-025-07542-w)
Supplement: Supplementary file 2 — Supplementary materials [file 42003_2025_7542_MOESM2_ESM.pdf]

Supplementary materials

This file contains:

Supplementary data S1-5 (provided as separate excel files)

Supplementary figures S1-S5

Supplementary tables

Supplement data S1 Biogeochemical and environmental data from sampling stations. NA = not available.

Supplement data S2 Particle-associated Gammaproteobacteria N_2 fixation rates in different particle size classes.

Supplement data S3 Particle-associated putative NCD N_2 fixation rates in different particle size classes.

Supplement data S4 Normalised ASV counts and taxonomy.

Supplement data S5 Particle-associated N_2 fixation rates from each station and cell

Supplementary figures

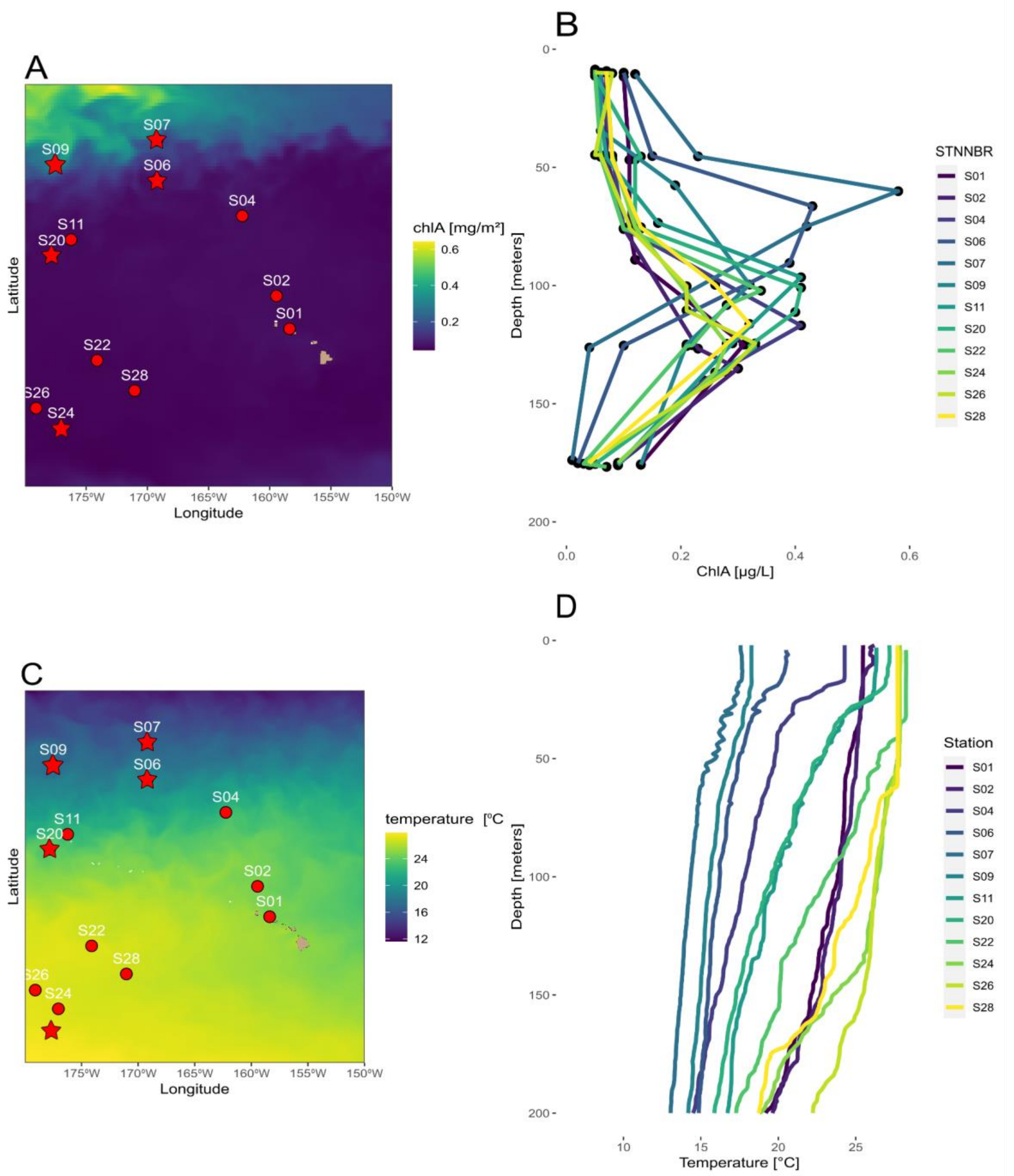

Figure S1 Sampling stations during the NCD cruise (KM2206). A and B show surface and water column chlorophyll *a* (Chl *a*) concentrations, respectively, C and D show surface and water column temperature, respectively. The data shown is a composite of the cruise duration (i.e., 4 June to 6 July 2022). The data in A and C were obtained from the E.U. Copernicus Marine Service information; DOI 10.48670/moi-00016 and DOI 10.48670/moi-00015. Stations selected for nanoSIMS are shown as a star.

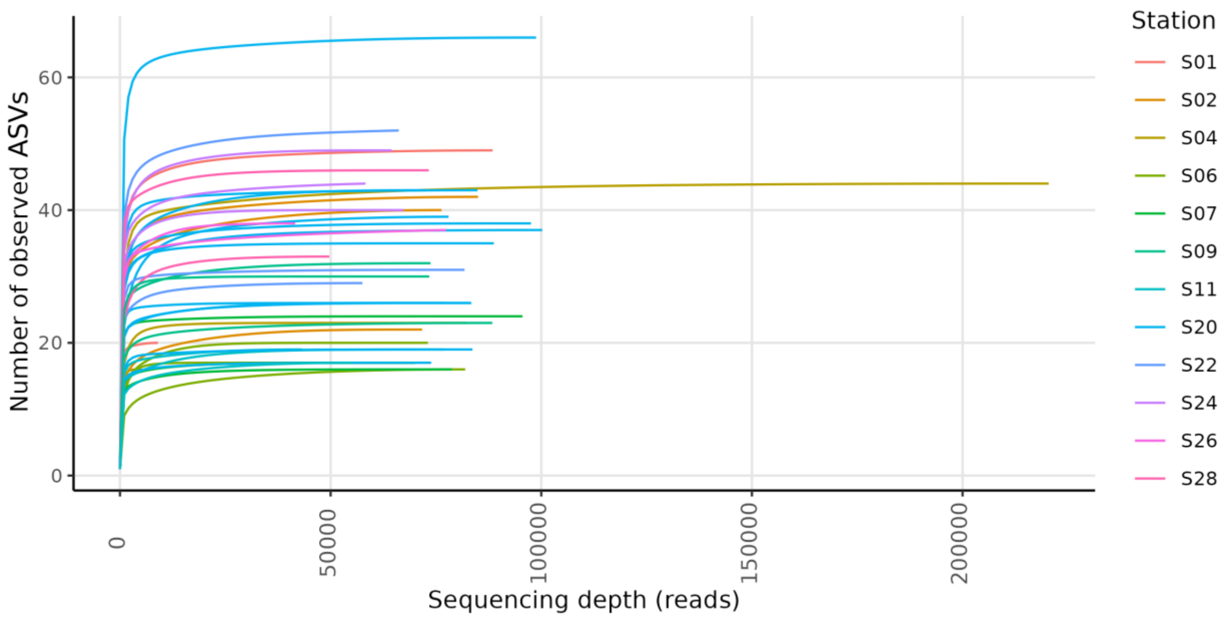

Figure S2 Rarefaction of amplicon sequencing data coloured by sampling station. Sequencing depth in reads on the x-axis, and number of amplicon sequence variants (ASVs) obtained on the y-axis.

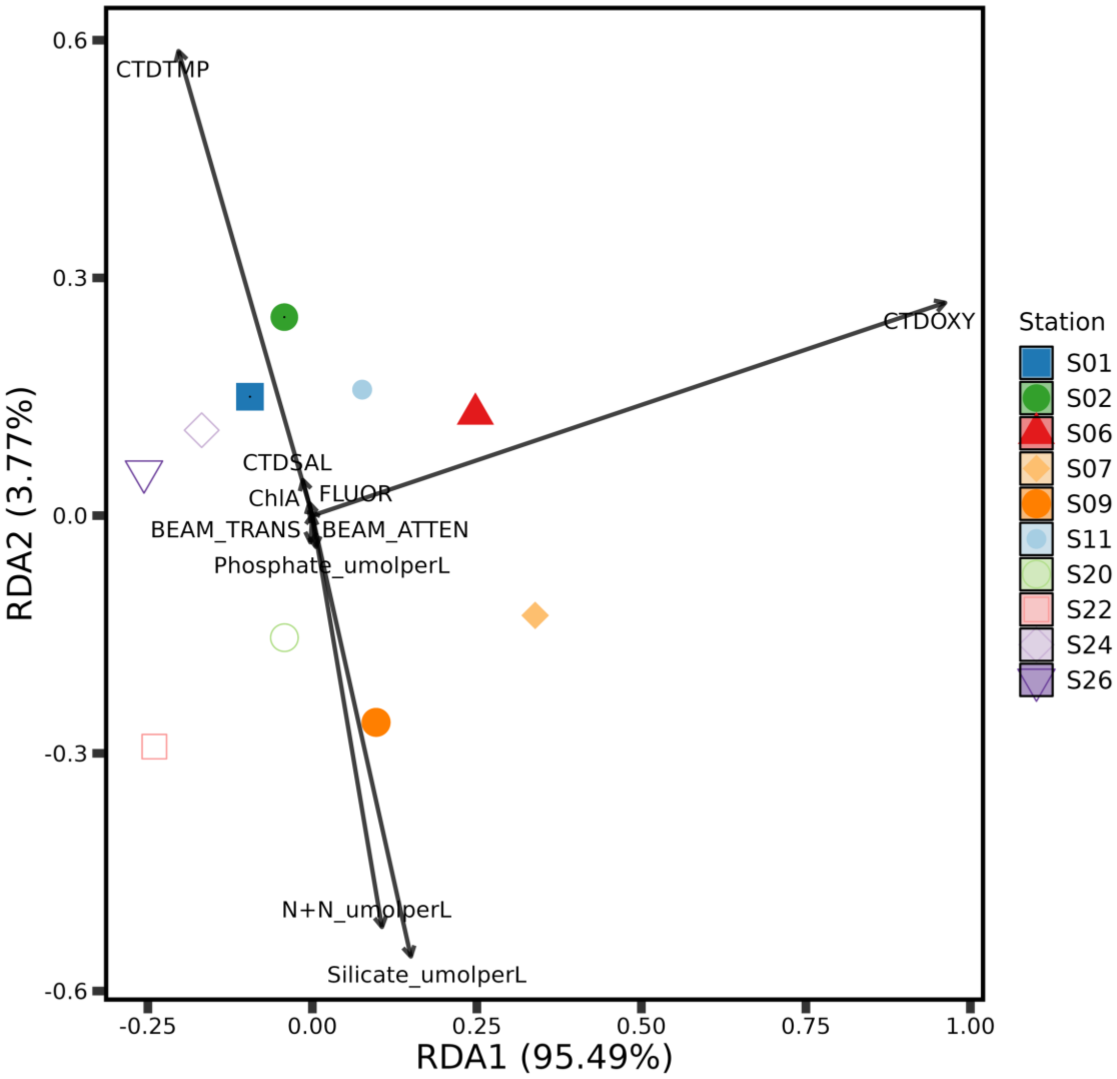

Figure S3 Redundancy analysis of environmental and biogeochemical factors across sampling stations. CTDTMP is temperature in ºC, CTDOXY is oxygen (µmol l^{-1}), BEAM_TRANS and BEAM_ATTEN is the beam transmission (%) and attenuation (m^{-1}), respectively. FLUOR is the fluorescence measured by the sensor attached to the CTD package, and chlA is the concentration of Chlorophyll-*a* measured from Niskin bottle samples (µg l^{-1}) water.

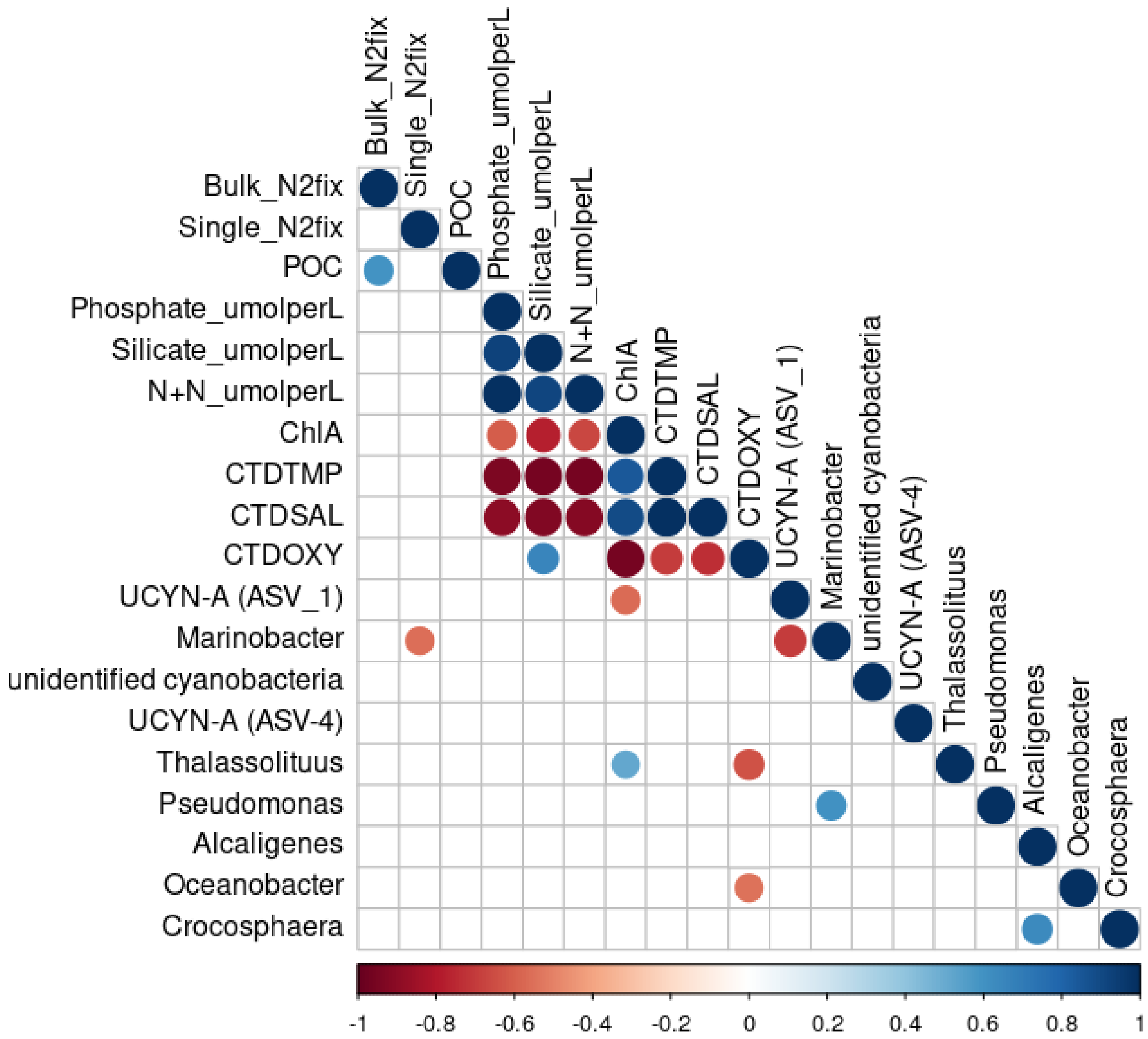

Figure S4 Correlation plot of biogeochemical, environmental data and the top ten amplicon sequence variants (ASVs) in station S06, S07, S09, S11, S20 and S24, from all MSC fractions. Blue indicates positive correlation and red indicates negative correlation. NCD are *Marinobacter*, *Thalassolituus*, *Pseudomonas*, *Alcaligenes and Oceanobacter*, respectively. Cyanophyceae are related to UCYN-A (ASV-1), unidentified cyanobacteria, UCYN-A (ASV-4) and *Crocosphaera*, respectively.

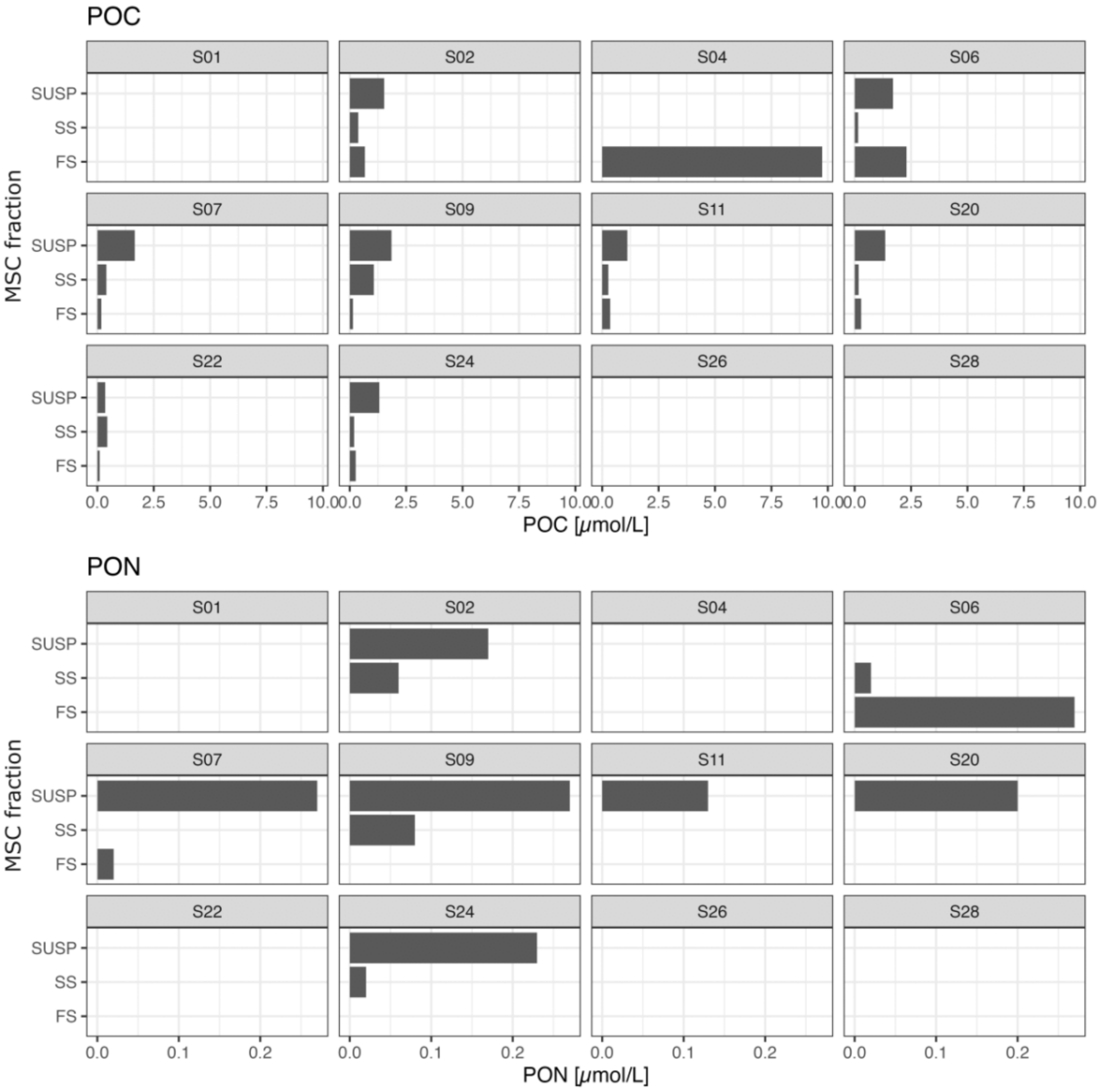

Figure S5 Particulate carbon (POC) and particulate nitrogen (PON) concentrations in different marine snow catcher (MSC) fractions and sampling stations.
