# Supplementary material for: Unveiling the contribution of particle-associated non-cyanobacterial diazotrophs to N2 fixation in the upper mesopelagic North Pacific Gyre
Source: Commun Biol. 2025 Feb 22;8:287. doi: 10.1038/s42003-025-07542-w (PMC11846875; doi:10.1038/s42003-025-07542-w)
Supplement: Supplementary file 3 — Description of Additional Supplementary Files [file 42003_2025_7542_MOESM3_ESM.pdf]

Description of Additional Supplementary Files

File name: Supplementary data S1

Description: Biogeochemical and environmental data from sampling stations. NA = not available.

File name: Supplementary data S2

Description: Particle-associated Gammaproteobacteria N2 fixation rates in different particle size classes.

File name: Supplementary data S3

Description: Particle-associated putative NCD N2 fixation rates in different particle size classes.

File name: Supplementary data S4

Description: Normalised ASV counts and taxonomy. Supplement data S5 Particle-associated N2 fixation rates from each station and cell.

File name: Supplementary data S5

Description: Particle-associated N2 fixation rates from each station and cell.
